# Trehalose alleviates salt tolerance by improving photosynthetic performance and maintaining mineral ion homeostasis in tomato plants

**DOI:** 10.3389/fpls.2022.974507

**Published:** 2022-08-12

**Authors:** Yan Yang, Jianming Xie, Jing Li, Jing Zhang, Xiaodan Zhang, Yandong Yao, Cheng Wang, Tianhang Niu, Emily Patience Bakpa

**Affiliations:** College of Horticulture, Gansu Agricultural University, Lanzhou, China

**Keywords:** trehalose, salt stress, photosystem fluorescence, OJIP transient, ionic homeostasis, tomato

## Abstract

Trehalose (Tre), which was an osmoprotective or stabilizing molecule, played a protective role against different abiotic stresses in plants and showed remarkable perspectives in salt stress. In this study, the potential role of Tre in improving the resistance to salt stress in tomato plants was investigated. Tomato plants (Micro Tom) were treated with Hoagland nutrient solution (CK), 10 mM Tre (T), 150 mM sodium chloride (NaCl, S), and 10 mM Tre+150 mM NaCl (S+T) for 5 days. Our results showed that foliar application of Tre alleviated the inhibition of tomato plant growth under salt stress. In addition, salt stress decreased the values of net photosynthetic rate (Pn, 85.99%), stomata conductance (gs, 57.3%), and transpiration rate (Tr, 47.97%), but increased that of intercellular carbon dioxide concentration (Ci, 26.25%). However, exogenous application of Tre significantly increased photosynthetic efficiency, increased the activity of Calvin cycle enzymes [ribulose diphosphate carboxylase/oxygenase (Rubisco), fructose-1,6-bisphosphate aldolase (FBA), fructose-1, 6-bisphosphatase (FBPase), glyceraldehyde-3-phosphate dehydrogenase (GAPDH), and transketolase (TK)], up-regulated the expression of genes encoding enzymes, induced stomatal opening, and alleviated salt-induced damage to the chloroplast membrane and structure. In the saline environment, photosynthetic electron transport was restricted, resulting the J-I-P phase to decrease. At the same time, the absorption, capture, and transport energies per excited cross-section and per active reaction center decreased, and the dissipation energy increased. Conversely, Tre reversed these values and enhanced the photosystem response to salt stress by protecting the photosynthetic electron transport system. In addition, foliage application with Tre significantly increased the potassium to sodium transport selectivity ratio (S_*K–Na*_) by 16.08%, and increased the levels of other ions to varying degrees. Principal component analysis (PCA) analysis showed that exogenous Tre could change the distribution of elements in different organs and affect the expressions of *SlSOS1*, *SlNHX*, *SlHKT1.1*, *SlVHA*, and *SlHA-A* at the transcriptional level under salt stress, thereby maintaining ion homeostasis. This study demonstrated that Tre was involved in the process of mitigating salt stress toxicity in tomato plants and provided specific insights into the effectiveness of Tre in mediating salt tolerance.

## Introduction

Recently, nearly 7% of agricultural land has been affected by salinization because of the anthropogenic activities, including irrigation practices, fertilizer application methods, industrial pollution, and climate change (Hashem et al., 2018; Yin et al., 2021). As a result, soil salinization has become a global ecological problem threatening the environment. It affects the sustainable development of agriculture and hinders and constraints agricultural development (Zamani et al., 2019; Guo et al., 2020). The high salt concentration absorbed by plant roots resulted in high osmotic potential, which caused the increase in sodium ions (Na^+^) and chloride ions (Cl^–^), exceeding the threshold level, impaired the structure of plant cell membrane, inhibited photosynthesis, produced toxic metabolites, and reduced nutrient absorption, thus hindering plant growth and productivity and causing death in serious cases (Wu et al., 2018a; Zamani et al., 2019; Bhattarai et al., 2021). Tanveer et al. (2020) found that salinity not only delayed the germination stage of tomato, but also reduced the plant growth, such as root and stem length, plant dry weight and fresh weight, leaf area, etc. Reportedly, the growth and development of plants under salt stress were related to photosynthetic performance and the carbon cycle (Asrar et al., 2017). Moreover, Goussi et al. (2018) found that high concentration of salt stress led to thylakoid expansion and starch accumulation, which affected the donor side of PSII and reduced the quantum yield of PSI. Sodium chloride (NaCl) treatment diminished the stomatal aperture of tomato leaves, decreased net photosynthetic rate (Pn) and stomata conductance (gs), and inhibited chlorophyll synthesis (Faizan et al., 2021; Hu et al., 2021). In addition, long-term salinity reduced the activity of ribulose diphosphate carboxylase/oxygenase (Rubisco) and ribulose-1,5-bisphosphate (RuBP), thereby altering the biochemical reactions that regulate stomatal exchange (Loudari et al., 2020). Subsequently, the ability of several parts of the photosynthetic electron transport process in tomato seedlings was obstructed by the salt environment. Plants adapt to salt stress and mitigate its adverse effects through various methods and physiological mechanisms. For example, excess Na^+^ is compartmentalized into vacuoles and/or transported by apoplast pathways (Hashem et al., 2018). Loudari et al. (2020) showed that sodium (Na) was significantly enriched in the roots and copper (Cu) was greatly enriched in the aboveground of tomato plants, which affected the imbalance of ion homeostasis under salt stress. Salinity increased potassium ions (K^+^) efflux and decreased hydrogen ions (H^+^) efflux from tomato plants, resulting in a lower K^+^/Na^+^ ratio (Meng et al., 2020). Furthermore, salt stress significantly increased the transcriptional regulation of tonoplast Na^+^/H^+^ antiporter genes (Abdelaziz et al., 2019). In addition, plants exposed to salt stress synthesize and accumulate large amounts of compatible osmolytes, such as free amino acids, glycine betaine, and soluble sugars, to maintain their normal growth (Hashem et al., 2015; Farooq et al., 2017). However, not all sugars are considered involved in plant stress response. Glucose, fructose, sucrose, and trehalose (Tre) have been involved in the regulation of plant growth and development of the environmental response process (Lastdrager et al., 2014; Sehar et al., 2019).

Tre is an important and non-reducing disaccharide widely existing in organisms, and it can be used as an effective cytoprotective agent under extreme environments. In plants, the Tre synthesis pathway is the trehalose 6-phosphate (T6P) by trehalose-6-phosphate synthase (TPS) catalyzed the glucose 6-phosphate (G6P) and uridine diphosphate glucose (UDPG), and T6P was converted to Tre by trehalose-6-phosphate phosphatase (TPP) (Nunes et al., 2013). Ambastha and Tiwari (2015) observed that the desert plant accumulated high levels of Tre, suggesting that Tre played an essential role in response to abiotic stress. This special function of Tre may be related to its physical and chemical properties. Reportedly, Tre can prevent the deformation of protein and membrane structures by replacing polar hydrogen-bonding groups of water molecules (Avonce et al., 2006). Therefore, Tre has been used as a tool to interfere with plant processes to better address plant growth and development mechanisms with the advantages of being available, absorbable, cheap, and non-toxic (Lin et al., 2017). Hossain et al. (2019) suggested that the improvement of Tre mediated abiotic stress may be related to the activation of stress responsive genes and transcription factors, rather than as an osmoprotective molecule. Overexpression of *OsTPS8* enhanced salt tolerance without any yield reduction, indicating its improvement in rice genetics (Sarkar and Sadhukhan, 2022). Similarly, a number of studies have confirmed this. Krasensky et al. (2014) found that *AtTPPD*-overexpressing plants were more tolerant of salt stress than wild-type Arabidopsis thaliana plants. Depletion mutations in TPPF, a member of the TPP gene family in Arabidopsis thaliana, lead to a drought-sensitive phenotype, while *AtTPPF* seedlings upregulated the gene expression of drought-related electron transport activity and cell wall modification and downregulated stress-related transcription factors associated with water deficit (Lin et al., 2019). *AtTPPE* and *AtTPPI* were regulated by ABA response element binding factors, which directly bind to their promoters to enhance the gene expression, leading to increased Tre levels and a rapid response to stress (Lin et al., 2020; Wang et al., 2020). The overexpression of *ClTPS3* in Arabidopsis thaliana significantly improved salt tolerance by increasing the Tre content, enhancing the activities of superoxide dismutase (SOD) and peroxidase (POD) (Yang et al., 2022). In addition, transgenic plants overexpressing certain TPS/TPP genes from bacterial, fungal, or plants can improve the tolerance to abiotic stresses. Occasionally, these processes are accompanied by elevated Tre levels (Lyu et al., 2013; Henry et al., 2015; Lin et al., 2020; Wang et al., 2020). Tre reduced the effects of NaCl on growth parameters, Na, K, K/Na ratio, phenolics, and the expression of AOX, NHX1, and SOS1, thereby alleviating the adverse effects of NaCl in wheat (Samadi et al., 2019). Interestingly, T6P is considered a signaling metabolite that regulates sucrose levels (Figueroa and Lunn, 2016). Yadav et al. (2014) considered the T6P: sucrose ratio to be a key parameter in plants, which forms a homeostatic mechanism that maintains sucrose levels within a range appropriate to cell types and developmental stages. The sucrose-T6P model proposes that high levels of sucrose are accompanied by high levels of T6P, but that high levels of T6P may have positive or negative consequences for growth, depending on the metabolic environment and sucrose levels (Figueroa and Lunn, 2016; Fichtner et al., 2021). The sucrose-T6P relationship also modulates with low temperature (Figueroa et al., 2016). Kretzschmar et al. (2015) confirmed that *OsTPP7* promoted the transcription of MYBS1 and CIPK15 genes by regulating T6P: sucrose in germination tissues, thereby promoting anaerobic germination tolerance. There, research has shown that T6P promoted starch synthesis through the thioredoxin-mediated triggering of AGPase. T6P controlled starch hydrolysis in plastids and it could be transformed to Tre (Sadak, 2019). Exogenous Tre or Tre derivatives increased the maximum electron rate, maximum light use efficiency and minimum saturating irradiance of maize inhibited by water deficit stress (de Novais Portugal et al., 2021). In another study, application of Tre alleviated the negative effects of drought by partially restoring chlorophyll levels and photosynthetic activity in Sweet Basil (Zulfiqar et al., 2021). In addition, the application of Tre not only alleviated the heat-induced chlorophyll content and gas exchange parameters of *Emmenopterys henryi* Oliv., but also promoted the recovery of photosynthesis after heat treatment (Feng et al., 2022). Alternatively, Tre decreased the Na^+^/K^+^ ratio of rice seedlings under salt stress (Nounjan et al., 2012). In Arabidopsis thaliana, Tre also has the ability to retain K and K/Na, thus improving salt tolerance of plants (Yang et al., 2014). Altogether, these data strongly suggest that Tre plays a central role in stress.

Tomato (*Solanum lycopersicum*), which belongs to *Solanaceae*, is one of the important economic crops globally and has worldwide distribution (Zhong et al., 2019). To date, although the effects of Tre on salt stress have attracted extensive attention, most studies have concentrated on gas exchange parameters and K^+^ and Na^+^ content. However, a limited number of researches focused on the photosynthetic electron transport chain, distribution of different mineral elements, and the interaction between photosynthesis and ion transport under salt stress. The purpose of this study was to verify the hypothesis that exogenous Tre could affect the absorption and distribution of mineral elements in tomato plants, and alter the photosynthetic electron transport chain to improve plant salt tolerance. The positive effects of Tre were investigated by measuring the growth parameters, stomatal morphology, chloroplast ultrastructure, gas exchange parameters, Calvin cycle, photosynthetic electron transport chain, mineral element content, and ion transport related genes of tomato plants under salt stress. Therefore, a primary objective of this study is to determine whether Tre has a protective function on the photosynthetic mechanism and whether it can coordinate the beneficial distribution of different mineral elements in salt stress.

## Materials and methods

### Plant growth conditions

The experiment was carried out in plant growth chambers (RDN, Nibo) with a humidity of 70%, an ambient temperature of 26 ± 1°C during the day/20 ± 1°C at night, and photoperiod of 16 h/day. Germinated seeds of tomato (Micro-tom; Supplementary Figure 1) were sown in a tray with vermiculite and perlite. At the one-leaf stage, soilless-tray seedlings of similar size were transferred to plastic containers containing half-concentrated Hoagland’s nutrient solution. After 5 days of adaptation to hydroponics, tomato seedlings were grown with whole Hoagland’s nutrient solution, which was replaced every 5 days.

### Experimental design and treatment

When the fourth real-leaf of the seedlings fully expanded (25–30 days after sowing), the experimental treatment was started. The experiment adopted a completely randomized design with four treatments. Three biological replicates of 40 plants each were set for each treatment, with a total of 120 seedlings per treatment.

The treatment combinations were as follows: normal conditions (CK, Hoagland nutrient solution); normal conditions + Tre (T, Hoagland nutrient solution, 10 mM Tre); salt conditions (S, 150 mM NaCl); salt conditions + Tre (S+T, 150 mM NaCl, 10 mM Tre). The formulation of the Hogeland nutrient solution was shown in Supplementary Table 1. Tre solution was sprayed on the leaf surface of tomato plants under T and S+T treatments until the water drops were about to drip, continuously for 2 days. Similarly, CK and S treatments were sprayed with the same volume of pure water (2 mL/plant/spray; between 8:00 and 9:00 a.m.). Then, the seedlings were exposed to either a normal or excess NaCl condition. After 5 days of salt treatment, the tomato seedlings were randomly sampled for physiological and genetic data analyses. Functional leaves (fully expanding the functional leaves from the bottom to the fourth or fifth branches) were collected to measure stomatal characteristics, chloroplast ultrastructure, photosynthetic gas exchange parameters, Calvin cycle key enzymes and genes, rapid fluorescence determination of chlorophyll, and JIP-test. Roots, stems and leaves of tomato plants were isolated to measure mineral element content and relative expression levels of ion transport genes. The concentrations of NaCl and exogenous Tre used in this study were referred to our previous studies (Yang et al., 2022).

### Plant height and stem diameter

Plant height: the distance from the base of the stem to the growing point was measured using a scale (three tomato plants per treatment, *n* = 3). Stem thickness: the diameter of the hypocotyl at the base of the cotyledons was measured using vernier calipers (three tomato plants per treatment, *n* = 3).

### Fresh weight and dry weight

The shoot and root of the tomato plants (three tomato plants per treatment, *n* = 3) were separated with a sterile scalpel and weighed separately for fresh weight using an analytical balance. Then, the samples were placed in an oven at 80°C to be dried to constant weight, and their dry weight was recorded.

### Leaf area

Leaf images (three tomato plants per treatment, *n* = 3) were scanned with the Epson Expression 11000XL. The total leaf area of tomato plants was calculated using WinRHIZO software (WinRHIZO Pro LA2400, Canada).

### Scanning electron microscopy

The fresh leaves (three tomato plants per treatment, *n* = 3) were immediately fixed by electron microscopy fixative for 2 h at room temperature and then transferred to 4°C for preservation. The samples were washed with 0.1 M phosphate buffer saline (PBS, pH = 6.8) for four times with each wash lasting for 10 min. Then, the leaf samples were dehydrated with different concentrations of ethanol (30, 40, 50, 60, 65, 70, 75, 80, 85, 90, 95%), for 20 min each dehydration. Afterward, the samples were rinsed thrice with absolute ethanol, for 30 min each session. Finally, the samples were transferred to different concentrations of tert-butanol (30, 50, 70, 80, 85, 90, 95, 100, 100, 100%), with each transfer lasting for 30 min. Dry specimens were attached to metallic stubs using carbon stickers and sputter-coated with gold. The images were observed and captured using scanning electron microscope (SEM, S-3400N, Hitachi, Tokyo, Japan) (Min et al., 2019).

### Ultrastructure observation

The fresh leaves (three tomato plants per treatment, *n* = 3) were immediately fixed by electron microscopy fixative for 2 h at room temperature and transferred to 4°C for preservation. Then, the tissues were washed thrice using 0.1 M PBS (pH = 7.4), and each wash lasted for 15 min. Tissues that avoided the light were post-fixed with 1% osmic acid (OsO_4_) in 0.1 M PBS (pH = 7.4) for 7 h at room temperature. After removing OsO_4_, the tissues were rinsed thrice with 0.1 M PBS (pH = 7.4), with each rinse lasting for 15 min. Then, the plant tissues were gradient dehydrated by ethanol, permeated by acetone, embedded by 812 embedding agent, and polymerized in an oven. The resin blocks were cut to 60–80 nm thickness on an ultra-microtome (Leica UC7, Germany), and the tissues were fished out onto the 150 meshes cuprum grids with a formvar film. Then, they were stained with 2% uranium acetate and 2.6% lead citrate solution and observed by transmission electron microscope (TEM, HT7800, Hitachi, Japan) for image analysis. Samples preparation was completed by Wuhan Servicebio Biological Information Technology Co., Ltd.

### Gas exchange parameters

Intercellular carbon dioxide concentration (Ci), transpiration rate (Tr), gs, and Pn of tomato leaves were measured from 9:00 to 10:30 a.m. by a portable photosynthetic system (CIRAS-2, United Kingdom). The fourth or fifth fresh leaves of tomato plant were collected for measurement (three tomato plants per treatment, *n* = 3). The conditions set during measurement were as follows: leaf area, 1.7 cm^2^; chamber flow rate, 200 ml min^–1^; photosynthetically active irradiation, 1,000 μmol m^–2^ s^–1^; carbon dioxide (CO_2_) concentration, 400 μmol mol^–1^; air temperature, 25°C; and relative humidity, 60% (Wu et al., 2018b).

### Calvin cycle key enzymes

The activities of key enzymes of the Calvin cycle [Rubisco, fructose-1, 6-bisphosphatase (FBPase), transketolase (TK), glyceraldehyde-3-phosphate dehydrogenase (GAPDH), and fructose-1,6-bisphosphate aldolase (FBA)] were measured (three tomato plants per treatment, *n* = 3) using ELISA kits (Yaji Biotech, Shanghai, China). Leaf samples were thoroughly ground with extraction buffer (0.05 mM Tris-HCl, and 0.1 M phosphate buffer; pH 7.4) and then centrifuged at 3,000 g for 15 min at 4°C. The supernatant was used for enzyme activity assay. Added testing sample, standard and horseradish peroxidase-conjugate reagent to microplate wells. The antibody to Rubisco was pre-existing in the microplate wells. Then incubated for 60 min at 37°C and washed. The substrate 3,3’,5,5’-tetramethylbenzidine was converted to blue under the catalysis of peroxidase and to the final yellow under the function of acid. The intensity of the color was measured at 450 nm using a Spectra Absorbance Reader (ABS, United States). Draw a standard curve of optical density versus Rubisco activity from the measured values of the standards. The activity of Rubisco in the samples was then determined by comparing the O.D. of samples to the standard curve. Similarly, the activities of other enzymes were also determined. Under optimal conditions, the amount of enzyme required to convert 1 μmol of substrate in 1 min is called an enzyme activity (U). U/L is the international unit of enzyme activity (U/L represents the enzyme activity per liter of enzyme preparation; U/mL represents the enzyme activity per milliliter of enzyme preparation).

### Rapid fluorescence determination of chlorophyll and kinetics analysis

Referring to Tang et al. (2021) and Yang et al. (2021a). The chlorophyll fluorescence kinetics (OJIP curve) in the tomato plants (three tomato plants per treatment, *n* = 3) was measured with a multifunctional plant efficiency analyzer (Handy PEA, Hansatech, United Kingdom). Before determination, the leaves were darkened for 30 min and then exposed to continuous illumination (3,000 μmol m^–2^s^–1^). The OJIP curve was plotted with the logarithm of the measurement time as the abscissa and instantaneous fluorescence of 10 μs to 2 s as the ordinate. The parameters of the JIP-test and their calculation formulas were shown in Supplementary Table 2. Each treatment measurement was replicated 12 times from randomly selected plants.

### Mineral ion content

In accordance with Li et al. (2020), the samples (three tomato plants per treatment, *n* = 3) were digested in concentrated hydrogen peroxide and sulfuric acid solution. The ion contents [Na, potassium (K), calcium (Ca), magnesium (Mg), iron (Fe), manganese (Mn), zinc (Zn), and Cu] in leaves, stems, and roots were measured using an atomic absorption spectrometer (ZEEnit 700P, Analytik Jena, Germany). Referring to the method of Wang et al. (2010), the K-Na transport selectivity ratio was calculated (S_*K–Na*_):


(1) $${\text{S}}_{\text{k-Na}}=\left\{{\left[{\text{K}}^{+}\right]}_{\text{stem and leaf}}/\left[{\text{Na}}^{+}\right]\text{stem and leaf}\right\}/\left\{{\left[{\text{K}}^{+}\right]}_{\text{root}}/{\left[{\text{Na}}^{+}\right]}_{\text{root}}\right\}$$

### Quantitative real-time PCR (qRT-pCR)

After 5 days of NaCl treatment, fresh samples from each treatment (three tomato plants per treatment, *n* = 3) were washed with sterile water and placed in liquid nitrogen immediately. The tomato plant total RNA was extracted using the *SteadyPure* plant RNA extraction kit AG21019 (Accurate Biotechnology Co., Ltd, Hunan, China). The total amount, purity and integrity of the total RNA of the sample were shown in Supplementary Table 4 and Supplementary Figure 2. The cDNA was synthesized using the *Evo M-MLV* RT Kit with gDNA Clean for qPCRII kit AG11711 (Accurate Biotechnology Co., Ltd, Hunan, China). Real-time PCR was performed using a Light Cycler^®^ 96 Real-Time PCR System (Roche, Switzerland), following the methods in the SYBR^®^ Green Premix *Pro Taq* HS qPCR Kit AG11701 (Accurate Biotechnology Co., Ltd, Hunan, China). Tomato actin was used as a reference gene to normalize the data. Shanghai Shenggong Bioengineering Co., Ltd synthesized all gene primer sequences as listed in Supplementary Table 3. The 2^–ΔΔ*Ct*^ method was used to calculate the relative expression data of mRNA (Livak and Schmittgen, 2001). Three biological replicates were set for each treatment.

### Statistical analysis

Microsoft Excel 2010 and SPSS 22.0 (SPSS Institute Inc., United States) were used for collation and counting, respectively. Duncan’s multiple range test (*p* < 0.05) was used to compare differences among various treatments. Principal component analysis (PCA) and other diagrams were compiled by OriginPro 2021 (OriginLab Institute Inc., United States). All experiments were performed in triplicate. The results were represented by mean values ± standard error.

## Results

### Effect of trehalose on growth attributes and growth parameters under salt stress

**FIGURE 1:**
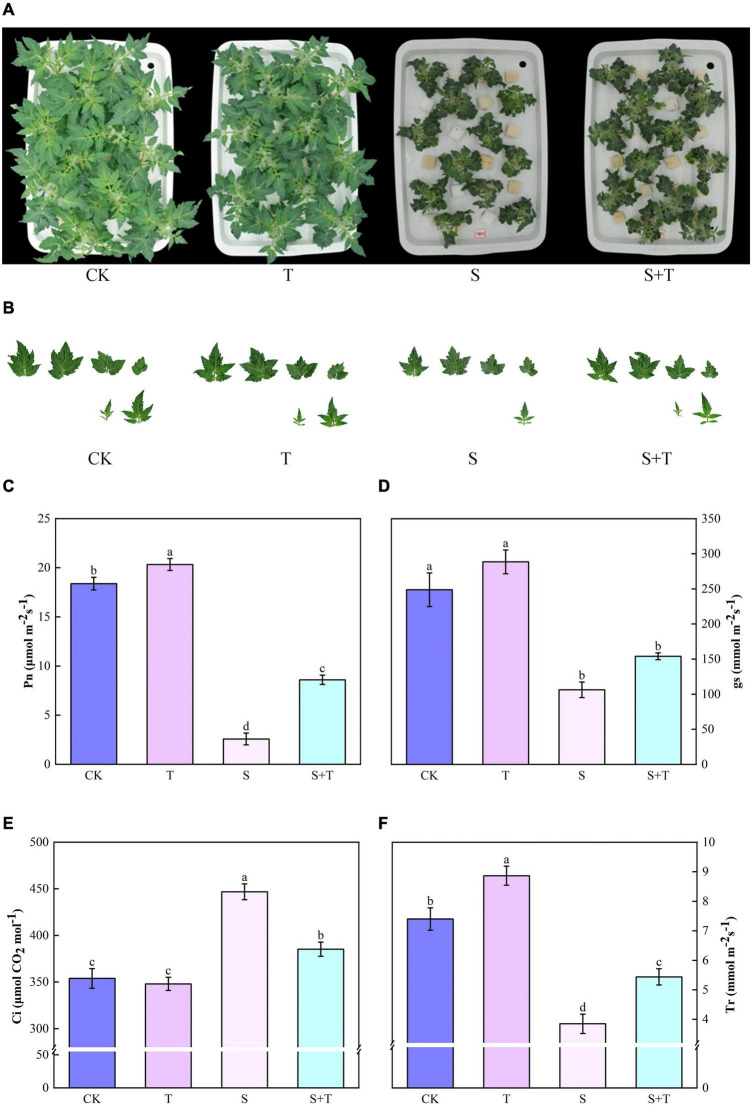
Effects of Tre treatment on growth attributes and photosynthetic gas exchange parameters of tomato seedlings exposed to salt stress for 5d. **(A)** Growth attributes. **(B)** Leaf area. **(C)** Net photosynthetic rate (Pn). **(D)** Stomata conductance (gs). **(E)** Intercellular carbon dioxide concentration (Ci). **(F)** Transpiration rate (Tr). The results showed the mean ± SE of three replicates, and the different letters denote the significant difference among treatments (*P* < 0.05), according to Duncan’s multiple tests. CK, control; T, 10 mM Tre; S, 150 mM NaCl; S+T, 150 mM NaCl + 10 mM Tre.

The phenotypes of tomato plants in each treatment at 5 d were observed and photographed (Figures 1A,B). The exogenous application of Tre had no evident effect on the plants cultivated in the normal nutrient solution. However, the leaves of the tomato plants treated with S and S+T, which inhibited the growth and development of tomato plants, were remarkably curled, and the color was dull. However, the wilting degree of leaves under S treatment was higher than that in S+T treatment. As shown in Table 1, S treatment significantly inhibited plant height, stem diameter, shoot fresh weight, shoot dry weight, root fresh weight, and leaf area compared to CK. There was no significant difference in stem diameter, root dry weight and fresh weight among CK, T and S+T treatments. Meanwhile, compared with S treatment, S+T treatment increased plant height, shoot fresh weight, shoot dry weight and leaf area by 13.73, 36.38, 35.22, and 17.89%, respectively. Thus, Tre might play promotive roles on growth in tomato seedlings under salt stress.

**TABLE 1:** Effects of Tre treatment on the growth parameters in tomato seedlings under salt stress for 5 days.

Treatment	Plant height (mm)	Stem diameter (mm)	Shoot fresh weight (g)	Shoot dry weight (g)	Root fresh weight (g)	Root dry weight (g)	Leaf area (cm^2^)
CK	88.358 ± 2.894a	3.286 ± 0.123a	4.233 ± 0.301a	0.44 ± 0.031a	1.187 ± 0.067a	0.068 ± 0.003a	54.508 ± 1.629a
T	76.304 ± 2.16b	3.253 ± 0.038a	3.583 ± 0.055b	0.365 ± 0.01b	1.093 ± 0.068ab	0.063 ± 0.004a	54.357 ± 1.338a
S	58.214 ± 1.139d	3.001 ± 0.02b	1.537 ± 0.051d	0.195 ± 0.006d	0.942 ± 0.045b	0.06 ± 0.002a	36.382 ± 1.485c
S+T	67.48 ± 0.168c	3.165 ± 0.065ab	2.416 ± 0.041c	0.301 ± 0.007c	1.046 ± 0.093ab	0.07 ± 0.006a	44.307 ± 1.068b

Data of tomato plants under saline condition were obtained after 5 days. The results showed the mean ± SE of three replicates, and the different letters denote the significant difference among treatments (*P* < 0.05), according to Duncan’s multiple tests. CK, control; T, 10 mM Tre; S, 150 mM NaCl; S+T, 150 mM NaCl + 10 mM Tre.

### Effect of trehalose on growth attributes, photosynthetic gas exchange parameters, calvin cycle key enzymes, and genes under salt stress

**FIGURE 2:**
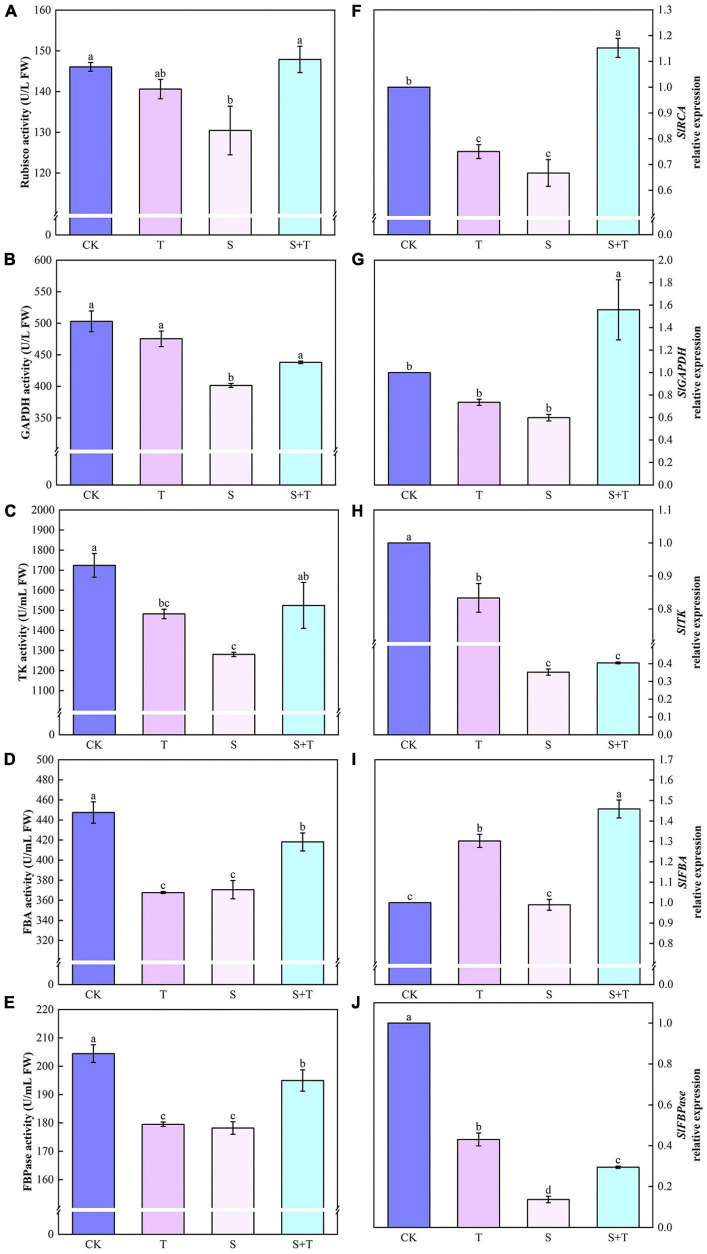
Effects of Tre treatment on Calvin cycle key enzymes and genes of tomato seedlings exposed to salt stress for 5 days. Panels **(A–E)** represent the activity of Rubisco, GAPDH, TK, FBA and FBPase, respectively. Panels **(F–J)** represent the expression levels of *SlRCA*, *SlGAPDH*, *SlTK*, *SlFBA*, and *SlFBPase*, respectively. The results showed the mean ± SE of three replicates, and the different letters denote the significant difference among treatments (*P* < 0.05), according to Duncan’s multiple tests. CK, control; T, 10 mM Tre; S, 150 mM NaCl; S+T, 150 mM NaCl + 10mM Tre.

Salt stress was associated with significantly lower Pn, gs, and Tr values than those of the control tomato plants at 5 days, whereas the reduction of these variables indicated that the exogenous Tre-pretreated plants were smaller than those under salt treatment (Figures 1B–D). However, the Ci of the tomato seedlings increased under NaCl treatment compared with the non-stressed control. The S+T treatment caused a significant decline (Figure 1E). As shown in Figure 2, NaCl treatment led to a significant decrease in Rubisco, FBA, FBPase, GAPDH, and TK activity and a downregulation in Calvin cycle-related genes. By contrast, exogenous Tre increased the activity of these enzymes. The genes involved in the Calvin cycle in the salt-stress tomato plants pretreated with Tre were primarily up-regulated.

### Effect of trehalose on stomatal characteristics and chloroplast ultrastructure under salt stress

**FIGURE 3:**
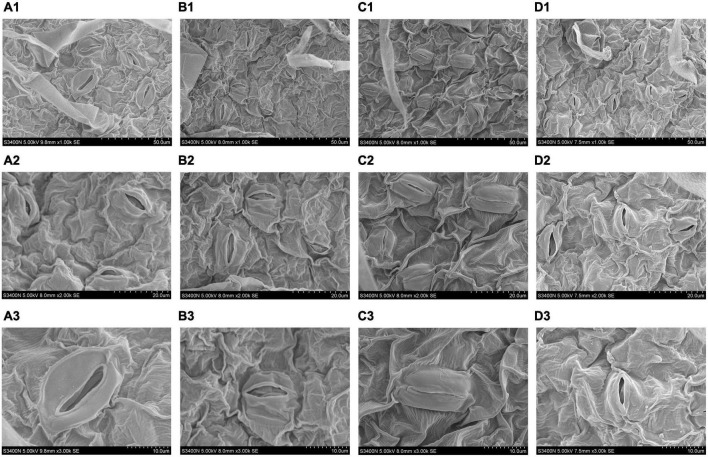
Effects of Tre treatment on stomatal characteristics of tomato seedlings exposed to salt stress for 5 days. Panels **(A1–D1)** represent stomatal distribution of tomato leaves in CK, T, S and S+T, respectively, 1,000×, scale bars = 50 μm; Panels **(A2–D2)** represent stomatal characteristics in CK-S+T, 2,000×, scale bars = 20 μm. Panels **(A3–D3)** represent stomatal characteristics in CK-S+T, 3,000×, scale bars = 10 μm.

Scanning electron microscope was used to observe the stomatal morphology of tomato leaves under different treatments. As shown in Figure 3, salt stress changed the stomatal opening. However, no significant structural differences were observed between Tre and no Tre treatments under normal conditions. After 5 days of salt stress, the closure degree of S treatment was higher than that of the S+T treatment. In addition, the stomata number of tomato leaves under the S treatment was lesser than that in the S+T treatment.

**FIGURE 4:**
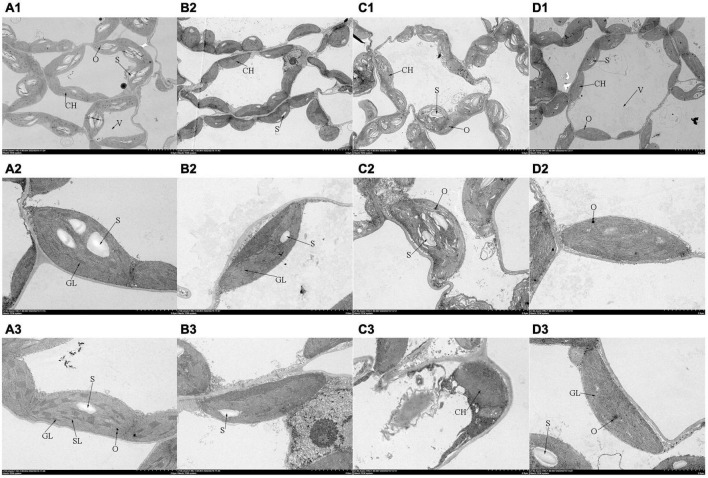
Effects of Tre treatment on the chloroplast ultrastructure of tomato seedlings exposed to salt stress for 5 days. Panels **(A–D)** represent the chloroplast ultrastructure of tomato leaves in CK, T, S, and S+T, respectively. **(A1–D1)**×2,000, scale bars = 5 μm; **(A2–D3)** ×7,000, scale bars = 2 μm. CH, chloroplast; V, vacuole; GL, grana lamellae; SL, stroma lamellae; S, starch grains; O, osmiophilic granules.

The chloroplast ultrastructure of leaves observed by TEM was shown in Figure 4. In the control plants, the chloroplasts possessed clear membrane structures and were ellipsoidal, the stromal thylakoids were tightly arranged, starch grains were packed, and osmiophilic granules were low in number. When subjected to salt stress, several prominent changes were observed in the chloroplast: (a) most cell membranes and chloroplast membranes were dissolved; (b) the chloroplast shape was swollen severely, (c) the thylakoid structure became vague with faults; (d) starch grains were reduced, and osmiophilic granules were increased. Compared with those under salt stress treatment, the number of osmiophilic granules decreased significantly with Tre addition, the status of the thylakoid membrane was optimized, and the number of grana and stroma lamellae increased. Interestingly, plasmolysis occurred in tomato leaves after the addition of Tre alone. In addition, the grana lamellae were loose, and the stroma lamellae were fuzzy.

### Effect of trehalose on the electron transfer of photosystem II under salt stress (OJIP and JIP-test analysis)

**FIGURE 5:**
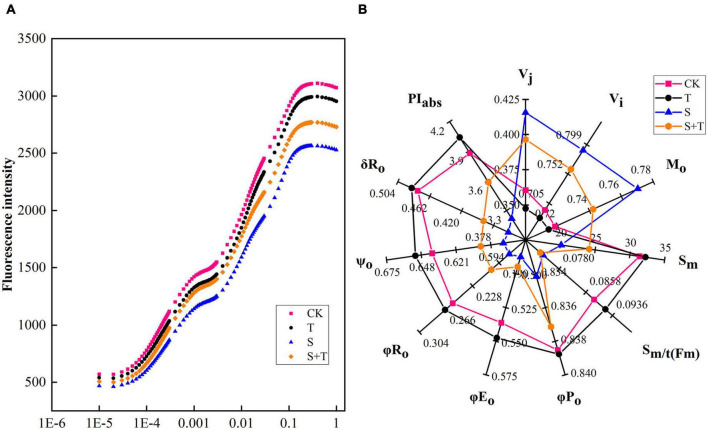
Effects of Tre treatment on chlorophyll fluorescence kinetics (OJIP curves) and JIP-text parameters of tomato seedlings exposed to salt stress for 5 days. **(A)** OJIP curves. **(B)** JIP-text parameters. The results showed the mean ± SE of three replicates. CK, control; T, 10 mM Tre; S, 150 mM NaCl; S+T, 150 mM NaCl + 10mM Tre.

To understand how Tre alleviated PSII photoinhibition induced by salt stress, we measured the OJIP curve of each treatment (Figure 5A). Experimental results showed no significant difference was found in the OJIP curve between the CK and T treatment. The J-I-P stage of the curve was significantly reduced by 5 days salt stress, compared with the normal control. However, the reduction in the J-I-P stage was more pronounced in S than in S+T treatment. In the radar representing various fluorescence parameters and performance indices, pronounced changes were observed in the tomato leaves when subjected to salt stress compared with the control seedlings (Figure 5B). In addition, Tre could effectively reverse the reduction of normalized total complementary area above the O-J-I-P (S_*m*_), maximum quantum yield for primary photochemistry (φP_*o*_), quantum yield for electron transport (φE_*o*_), the quantum yield of PSI final electron acceptor reduction per photon absorption (φR)_*o*_, probability that a trapped exciton moves an electron into the electron transport chain beyond Q_*A*_^–^ (ψ_*o*_), the efficiency of electron transfer from Q_*B*_ to PSI receptor (δR_*o*_), and performance index on absorption basis (PI_*abs*_), and promote the enhancement of relative variable fluorescence intensity at the J-step (V_*j*_), relative variable fluorescence intensity at the I-step (V_*i*_), and approximated initial slope of the fluorescence transient (M_*o*_) under salt stress, whereas no significant difference was observed in the average redox state of Q_*A*_ [S_*m/t(Fm*)_].

**FIGURE 6:**
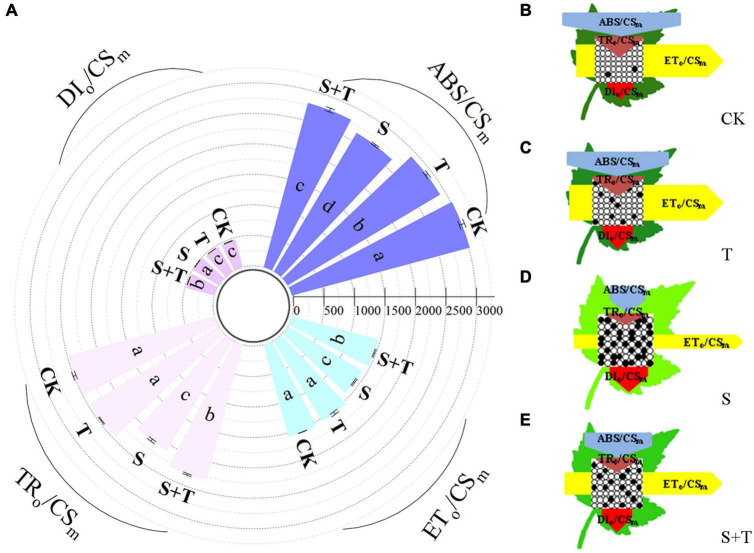
Effects of Tre treatment on phenomenological fluxes (per cross-section, CS_*m*_) of tomato seedlings exposed to salt stress for 5 d. **(A)** Electron absorption, capture, transport and dissipation flux per cross-section. The results showed the mean ± SE of three replicates, and the different letters denote the significant difference among treatments (*P* < 0.05), according to Duncan’s multiple tests. **(B–E)** Energy pipeline leaf model of phenomenological fluxes (per cross section, CS_*m*_) in CK, T, S, and S+T, respectively. Changes in each energy flux were indicated by resizing the corresponding arrows. Empty and full black circles showed the percentages of active and non-active reaction centers of photosystem II (PSII), respectively. CK, control; T, 10 mM Tre; S, 150 mM NaCl; S+T, 150 mM NaCl + 10 mM Tre.

**FIGURE 7:**
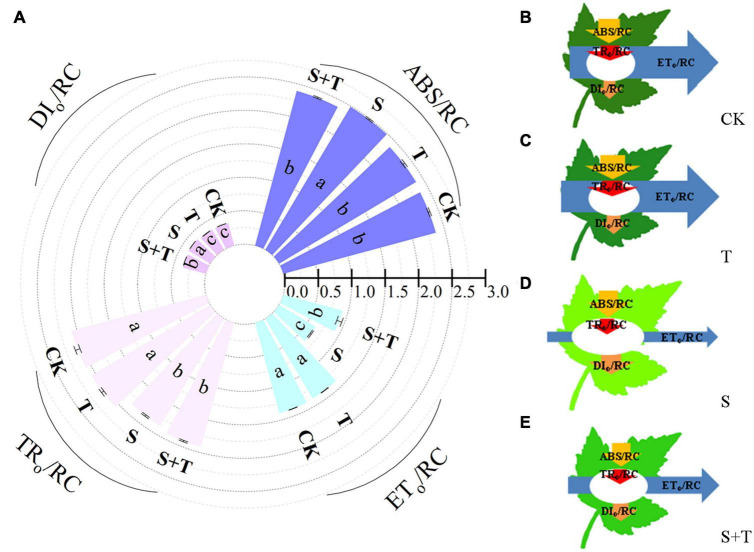
Effects of Tre treatment on phenomenological fluxes (per active reaction center, RC) of tomato seedlings exposed to salt stress for 5 d. **(A)** Electron absorption, capture, transport and dissipation flux per RC. The results showed the mean ± SE of three replicates, and the different letters denote the significant difference among treatments (*P* < 0.05), according to Duncan’s multiple tests. **(B–E)** Energy pipeline leaf model of phenomenological fluxes (per active reaction center, RC) in CK, T, S, and S+T, respectively. Changes in each energy flux were indicated by resizing the corresponding arrows. CK, control; T, 10 mM Tre; S, 150 mM NaCl; S+T, 150 mM NaCl + 10 mM Tre.

Based on the structural and functional parameters obtained by the JIP-test experiment, changes in the energy distribution per cross section and per active reaction center under different treatments were compared and analyzed to understand further the photosynthetic behavior of tomato leaves under different treatments (Figures 6, 7). The results showed that exogenous Tre had no effect on the energy distribution per cross section and per active reaction center of tomato plants growing under normal conditions. Under salt stress, the absorption flux per cross section (ABS/CS_*m*_), trapped energy flux per PSII cross section (TR_*o*_/CS_*m*_), and energy used for electron transport in PSII cross section (ET_*o*_/CS_*m*_) decreased significantly, whereas dissipated energy flux per PSII cross section (DI_*o*_/CS_*m*_) increased significantly. However, the application of exogenous Tre promoted the distribution trend of the above energy (ABS/CS_*m*_, TR_*o*_/CS_*m*_, and ET_*o*_/CS_*m*_) and reduced the heat dissipation under salt stress. In addition, the number of active reaction centers (RCs) per unit leaf area decreased. The light energy absorption flux per RC (ABS/RC) and dissipative energy flux per RC (DI_*o*_/RC) increased significantly after salt stress treatment, whereas, Tre application significantly inhibited these energy increases. In addition, salt stress reduced the trap energy flux per RC (TR_*o*_/RC) and excitation energies for the electron transport flux per RC (ET_*o*_/RC) in tomato leaves, while exogenous Tre reversed this trend. The membrane model visually showed the specific energy flux variation per cross section and per active reaction center (Figures 6B–E, 7B–E).

### Effect of trehalose on mineral ion content under salt stress

**FIGURE 8:**
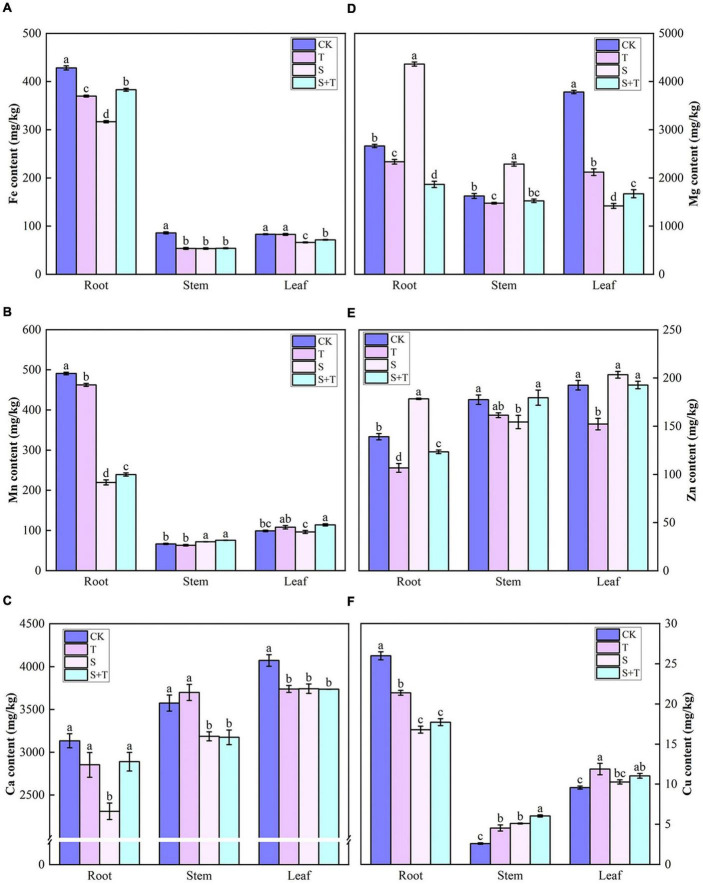
Effects of Tre treatment on mineral ion content in tomato seedlings under salt stress for 5 days. **(A)** Fe content. **(B)** Mn content. **(C)** Ca content. **(D)** Mg content. **(E)** Zn content. **(F)** Cu content. The results showed the mean ± SE of three replicates, and the different letters denote the significant difference among treatments (*P* < 0.05), according to Duncan’s multiple tests. CK, control; T, 10 mM Tre; S, 150 mM NaCl; S+T, 150 mM NaCl + 10 mM Tre.

The contents of six elements in the root, stem, and leaves of tomato under different treatments were demonstrated in Figure 8. Compared with the control group, the contents of Fe and Ca in the S-treated tomatoes were significantly reduced. Tomato roots contained more Fe, Mn, and Cu than stems and leaves for all treatments. Similarly, the leaves contained more Ca than stems and roots. The tomato plants treated with Tre exhibited unique ways of promoting the accumulation of mineral elements to varying degrees under salt stress. Compared with the S treatment, the S+T treatment promoted the accumulation of Mn and Fe in stems and leaves, Zn and Cu in stems, Mg in leaves, and Ca in roots.

### Variable interaction through principal component analysis revealed the effect of trehalose on ionomers under salt stress

**FIGURE 9:**
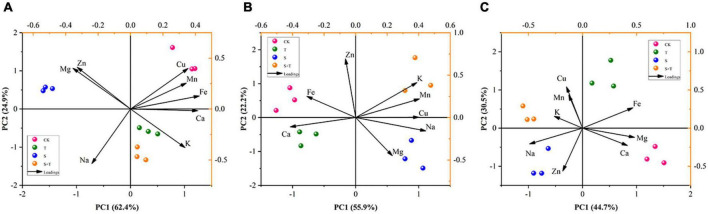
Principal component analysis (PCA) of tissue ionome variation in tomato under salt stress by Tre treatment, with the loadings of mineral elements to the PC1 and PC2. **(A)** Root ionome variation among treatments and the loadings of elements to the PC1 and PC2. **(B)** Stem ionome variation among treatments and the loadings of elements to the PC1 and PC2. **(C)** Leaf ionome variation among treatments and the loadings of elements to the PC1 and PC2. CK, control; T, 10 mM Tre; S, 150 mM NaCl; S+T, 150 mM NaCl + 10 mM Tre.

To thoroughly manifest the effect of Tre on mineral ions in different organs of tomato seedlings under salinity, we carried out PCA on previously measured mineral ions (Figure 9). PCA showed that the different treatments and organs of tomato plants could be distinguished. The first principal component (PC1) could be used to distinguish between S and S+T treatments, and represented 62.4, 55.9, and 44.7% of the total coefficient of variation in roots, stems, and leaves, respectively. In addition, the main contributing elements of PC1 were Ca and Fe in roots, Cu, Ca, and Na in stems, and K and Mg in leaves. The second principal component (PC2) could be used to distinguish between the salting and non-salting treatments, and accounted for 24.9, 22.2, and 30.5% of the total variance in roots, stems, and leaves, respectively. Subsequently, Na in roots, Zn in stems, and Mn and Cu in leaves had strong loading with the PC2, and they could be used as key elements for salt tolerance to reflect the difference in mineral elements in various organs of tomato plants.

### Trehalose regulated S_*K–Na*_ parameters and the expression of ion transport-related genes under salt stress

**FIGURE 10:**
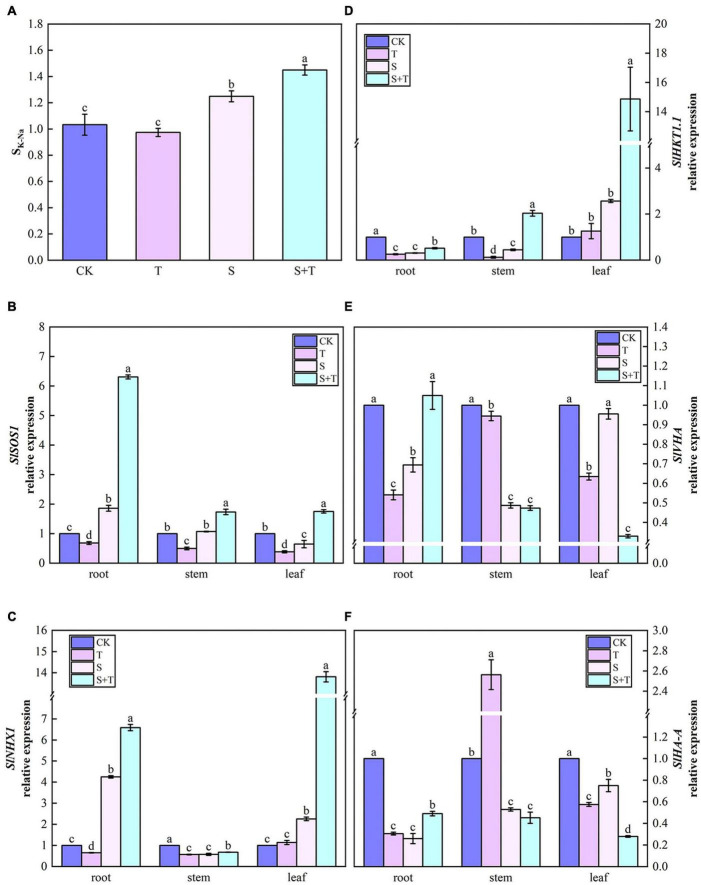
Effects of Tre treatment on the K-Na transport selectivity ratio and the relative expression of ion transport genes in tomato seedlings under salt stress for 5 days. Panel **(A)** represents the K-Na transport selectivity ratio (S_*K–Na*_); Panels **(B–F)** represent the expression levels of *SlSOS1*, *SlNHX1*, *SlHKT1.1*, *SlVHA*, and *SlHA-A*, respectively. The results showed the mean ± SE of three replicates, and the different letters denote the significant difference among treatments (*P* < 0.05), according to Duncan’s multiple tests. CK, control; T, 10 mM Tre; S, 150 mM NaCl; S+T, 150 mM NaCl + 10 mM Tre.

As shown in Figure 10A, at the salinity treatment level, the S_*K–Na*_ value of tomato plants increased by 20.96% compared with the control treatment. After the exogenous addition of Tre, the selectivity coefficients of K and Na transport from tomato plant roots to shoots significantly improved. The lowest S_*K–Na*_ was observed when Tre was sprayed alone under normal conditions. Then, we checked the expression levels of genes associated with Na^+^ and/or K^+^ transport (Figures 10B–F). Compared with control, the relative expression levels of *SlSOS1* in roots, *SlHKT1.1* in leaves, and *SlNHX1* in roots and leaves were significantly up-regulated at moderate salinity level. In addition, *SlSOS1* and *SlNHX1* genes were abundantly expressed in roots, whereas, *SlHKT1.1* was mostly transcribed in leaves. By contrast, *SlVHA* and *SlHA-A* were significantly downregulated in plants. Compared with salt-stressed tomato seedlings, the transcripts of all genes were significantly higher when Tre was applied with NaCl, except for *SlVHA* and *SlHA-A* in stems and leaves.

## Discussion

Salt stress obstructs plant growth and development, resulting in a loss of plant productivity. Previous studies demonstrated that Tre was critical in maintaining the growth development and osmotic equilibrium in plants exposed to salt stress (Sarkar and Sadhukhan, 2022; Yang et al., 2022). Tre treatment at 30 mM alleviated the inhibitory effects of NaCl on strawberry shoot growth (Samadi et al., 2019). Furthermore, Tre foliar treatments (10 and 50 mM) proved to be effective in increasing the value of the growth parameters (plant height, leaves number, fresh weight, and dry weight) and more accumulation of organic solutes of leaves (glucose, sucrose, Tre, and the soluble sugar) of salinity-stressed plants (Sadak, 2019). Tre ameliorated the responses of maize seedlings to salinity and alleviated the suppression of NaCl on shoot length, root length, and root volume (Rohman et al., 2019). In this work, NaCl treatment significantly inhibited tomato plants growth parameters (plant height, stem diameter, shoot fresh weight and dry weight, root fresh weight, and leaf area), whereas the application of Tre reversed this trend, thus increasing salinity tolerance (Table 1). Our result was in agreement with the findings of Abdallah et al. (2016) for rice, who indicated that Tre pretreatment resulted in the improvement of growth in plants exposed to salt stress. However, although Tre effectively enhanced the salt tolerance of tomato seedlings by increasing growth parameters, due to its complexity, the possible mechanisms need to be further studied.

Photosynthesis is a process sensitive to environmental stresses; for this, seeking a balance between the light energy absorbed by the photosystem and the energy consumed by metabolism is important for plant growth and development (Zhu et al., 2018). Numerous researchers have reported that salt stress could inhibit Pn, gs, and Tr parameters in plants (Tang et al., 2018; Castillejo-Morales et al., 2021; Yin et al., 2021). Our results showed that Pn, gs, and Tr decreased, and Ci increased under salt stress, which proved that the main factor of photosynthetic confinement was the non-stomatal limitation. Notably, the addition of exogenous Tre not only promoted the significant increase in Tr and Pn in tomato plants under normal growth conditions but also increased Pn and Tr and decreased Ci under salt stress (Figure 1). This was consistent with Feng et al. (2022). Under high temperature stress, Tre treatment increased Pn and Ci, but decreased gs and Tr, this differs from part of our results and may be that a Tre film was formed on the surface of leaves to reduce excessive evaporation of water and maintain water balance, thus inhibiting damage caused by high temperature (Zhao et al., 2019). In addition to the increased resistance of mesophyll cells to stomatal diffusion, decrease in CO_2_ solubility, and stability of photosynthetic machinery, affinity of Rubisco enzymes for CO_2_, and capability of RuBP to regenerate are important factors that led to the occurrence of non-stomatal factors (Castillejo-Morales et al., 2021; Pan et al., 2021). The Calvin cycle, as the final step of photosynthesis, is one of the non-stomata limiting factors, and it is the main pathway for plants to fix carbon (Ding et al., 2017; Sharma et al., 2020). Among them, Rubisco, GAPDH, and FBPase play important roles in carbon fixation, reduction, and RuBP regeneration stages, respectively (Niu and Ma, 2018; El Sayed et al., 2019; Wu et al., 2020). The enhancement of FBA and TK activity can promote the assimilation of CO_2_ in plant leaf tissues and the flow of carbon in the Calvin cycle, respectively (Lv et al., 2017). El Sayed et al. (2019) found that application of exogenous spermidine upregulated of Calvin Cycle related gene transcription levels in sweet sorghum seedlings under salt stress, which was similar to our results. We displayed that Tre promoted the increase in Rubisco, FBPase, FBA, GAPDH, and TK activities under salt stress and the up-regulation of related genes (Figure 2). This phenomenon might be explained by the adaptation to adversity, indicating that Tre pretreatment could alleviate the inhibitory effect of salt stress on the Calvin cycle by regulating the salinity-mediated accumulation of transcript of genes related to the Calvin cycle. In addition, an important response of plants to salt stress is to close the stomata, accompanied by a reduction in gs, which resulted to stomatal limitation of photosynthesis (Li et al., 2017). Niron et al. (2020) found that K^+^ was the main osmoregulatory substance of stomatal guard cells, coordinating gs, and Tr. In this study, the addition of exogenous Tre significantly alleviated the inhibition of photosynthesis by maintaining the stomatal aperture (Figure 3). Sucrose is a key product of photosynthesis in the majority of plants and excessive sucrose accumulation can diminish photosynthesis by inhibiting the expression levels of photosynthetic genes (Lunn et al., 2014; Sarkar and Sadhukhan, 2022). Fichtner and Lunn (2021) concluded that there was a high correlation between T6P (an intermediate of alginate) and sucrose levels, but the effect of T6P on sucrose was attributed to its inhibitory effect on the sucrose non-fermenting-1-related protein kinase1 (SnRK1). Degradation of SnRK1 is mediated by sumoylation and ubiquitination as well as intracellular oxidative regulation mediated by redox. More, SnRK1 is also a positive regulator of stomatal development (Han et al., 2020). bZIP11, another transcription factor that affects carbohydrate metabolism regulation, is also regulated by T6P. bZIPs play a regulatory role in developmental transition, carbohydrate and amino acid metabolism (Tsai and Gazzarrini, 2014). In summary, the application of a particular concentration of Tre improved photosynthesis, we hypothesized that the intermediate T6P, rather than Tre itself, may play a key regulatory signaling role in gas exchange parameters and carbon metabolism. In addition, *AtTPPI* and *AtTPPF* may be important mechanisms for coping with abiotic stresses by triggering ABA-mediated stomatal regulation (Lin et al., 2020; Wang et al., 2020).

Alternatively, chlorophyll fluorescence is a non-destructive, direct, and efficient method that has been widely used to evaluate the effects of abiotic stress on photosynthetic electron transport (Bednaøíková et al., 2020). Electron transport occurs primarily on the thylakoids of chloroplasts (Ma et al., 2018). At this point, salt stress caused separation of cytoplasmic wall and destruction of chloroplast structure, which was consistent with the results of Hameed et al. (2021) and Wu et al. (2021). Tre pretreatment could improve chloroplast damage of tomato plants under salt stress (Figure 4). Zushi and Matsuzoe (2017) assumed that salt stress may destroy the excited chlorophyll molecules in light-harvesting complex II, resulting in a low O-step value. Wang et al. (2021) indicated that the shape of the OJIP curve changed during NaCl stress, decreasing the I-P phase and increasing the J-I phase. In this research, the photochemical stage (O-J) and thermal phases (J-I and I-P) were affected by salt stress compared with the control, in which the J-I and I-P stages of the OJIP curve decreased under salt stress (Figure 5A). The possible reasons were as follows: on the one hand, salt stress reduced the reduction rate of the rapidly reducing PQ pool during the electron transfer, resulting in the decrease in I value. On the other hand, salt stress destroyed the chlorophyll proteins on the PSI acceptor side and/or reduced the number of RCs in PSII, thereby reducing the *P*-value (Oukarroum et al., 2018). By contrast, Goussi et al. (2018) displayed that moderate saline conditions had no significant effect on the rate of O-J phase and J-I phase in *Thellungiella salsuginea*. This finding contradicted our research results, and we believed that it could be attributed to the various salt tolerances of plants themselves. In addition, Tre inhibited the OJIP phases of tomato plants under salt stress. We speculated that Tre might play an active role in preventing the reduction of the QA pool size or the transfer of electrons from the PSII donor side. JIP-test can be used to identify energy absorption, trapping, and electron transport parameters in PSII and PSI and has been used in plant response to stress conditions (Zushi and Matsuzoe, 2017; Sameena and Puthur, 2021). Under salt stress, φE_*o*_ and φR_*o*_ decreased drastically, showing that salt stress damaged the primary photochemical reaction of tomato leaves and inhibited electron transfer on the PSII receptor side. Similar results have been found in other plant studies (Zhang et al., 2018; Çiçek et al., 2018; Lotfi et al., 2020). In addition, higher values of PI_*abs*_, φP_*o*_, φE_*o*_, ψE_*o*_, δR_*o*_, and φR_*o*_ were noted with S+T treatment than with S treatment, indicating that Tre treatment improved the photochemical efficiency (Figure 5B), electron transport flux ratio, and quantum efficiency of PSII to PSI under salt stress. PSII is one of the most sensitive components of photosynthesis, and abiotic stress can especially lead to the excessive reduction of electron transport chain (Lotfi et al., 2018). Our study showed that ABS/RC and DI_*o*_/RC values increased mainly, and TR_*o*_/RC and ET_*o*_/RC values markedly decreased under salt stress compared with the control. Such result was observed because salt stress caused the inactivation of most RCs, which decreased the energy trapping efficiency and electron transport efficiency from PSII (Zushi and Matsuzoe, 2017; Kiran and Mohan, 2021). Similarly, the analysis of energy allocation per unit cross-sectional area of tomato leaves under salt stress found a unique increase in DI_*o*_/CS_*m*_, whereas considerable decreases in ABS/CS_*m*_, TR_*o*_/CS_*m*_, and ET_*o*_/CS_*m*_ were observed in S treatment compared with the CK. In addition, Zuo et al. (2017) reported that nano-ZnO stress resulted in a decrease in TR_*o*_/CS_*m*_ and ET_*o*_/CS_*m*_ of wheat plants. Interestingly, we showed that Tre improved the energy absorption efficiency of PSII by suppressing the dissipation (DI_*o*_/CS_*m*_, DI_*o*_/RC) and increasing the trapped energy (TR_*o*_/CS_*m*_, TR_*o*_/RC), and electron transport flux (ET_*o*_/CS_*m*_, ET_*o*_/RC) per cross-section and per reaction center (Figures 6, 7). Both reducing subunits in Tre are used to form glycosidic bonds that can displace water and stabilize membranes in stress situations (MacIntyre et al., 2020). This suggestive of the fact that the unique structure of Tre at salinity may be the main reason for the protection of chloroplast structure. In contrast, the protection of Tre to the structure of thylakoid membrane can directly prevent the shedding of attached proteins, thus stabilizing the electron transfer function. Furthermore, protein complexes play an important role in linear electron transfer (Zhang et al., 2019). For example, the central role of CP43 and CP47 proteins is to receive excitation energy from the pigment complex of CP24, CP26, and CP29, and transfer the excitation energy to the pigment protein complex in the reaction center (Casazza et al., 2010; Zhang et al., 2010). However, excessive excitation energy limited production of reactive oxygen species (ROS), which leads to oxidative damage of the photosynthetic mechanism. Several studies have shown that Tre can enhance the activity of antioxidant enzymes in plants under abiotic stress, and regulate the expression of osmoprotectant biosynthetic genes such as proline (de Novais Portugal et al., 2021; Kosar et al., 2021; Zulfiqar et al., 2021; Feng et al., 2022). Therefore, the enhancement of ROS scavenging system induced by Tre may be one of the important reasons for the increase of photosynthetic oxygenation capacity and electron transfer rate of tomato seedlings under NaCl stress. In conclusion, Tre alleviated the photoinhibition of tomato leaves under salt stress.

The absorption of mineral elements by plants is conducive to growth and resistance to environmental stress (Ju et al., 2017; Liang et al., 2018). Ionic stress causes competition among ions for absorption, which will substantially affect the uptake and transport of mineral elements and ultimately cause mineral nutrient stress and imbalance of plant ion homeostasis (Guo et al., 2020). Previous studies have proven that salt stress reduces the content of mineral elements in rape (Naeem et al., 2010), cotton (Guo et al., 2019), wheat, and maize (El-Fouly et al., 2011; Iqbal et al., 2018). This could explain their lower growth rates leading to a reduction in biomass accumulation due to the negative effects of salt stress on assimilation capacity (Table 1). Moreover, salt stress prevented the uptake of nitrogen (N), phosphorus (P), Fe, Mn, Zn and other ions by plants, leading to symptoms of mineral deficiency (Ramireddy et al., 2018; Loudari et al., 2020). Zamani et al. (2019) found that micronutrient foliar sprays (Fe, Mn, Zn, and so on) can improve the salt tolerance of wheat; thus, changes in the mineral element contents are vital in the operation of resistance mechanism. Fe, Mg, and Mn are cofactors for multiple enzymes and have a pivotal position in chlorophyll biosynthesis (Ju et al., 2017; Zhang et al., 2017; Guo et al., 2020; Yang et al., 2021b). Mg concentration affects stomatal opening and can regulate the ionic currents across the chloroplast and vacuolar membrane (Misra et al., 2019). Recently, Yang et al. (2021b) discovered that Mn can reinforce plant cell walls, and such result was primarily attributed to the controlled formation of polysaccharides. In this experiment, salt stress suppressed the accumulation of Fe in the whole plant, Mg in leaves, and Mn in roots. By contrast, Guo et al. (2020) found that salt stress significantly increased the concentration of Mg in the roots and decreased in the leaves, while the concentrations of Fe and Mn in the leaves markedly increased, which was not completely consistent with our results. In our results, the content of Mg and Mn in leaves decreased, which may be caused by the damage of chlorophyll biosynthesis pathway under salt stress. Exogenous Tre facilitated the transport of Fe, Mg, and Mn from roots to their collection in leaves (Figure 8). This result may be one of the effective measures for Tre to maintain growth by chlorophyll synthesis, thereby enhancing photosynthesis to cope with salt stress (Figure 1). Zn is involved in stomatal opening and biotin synthesis. Meanwhile, it is also an essential element for the structural integrity of cell membranes (Tekdal and Cetiner, 2018; Misra et al., 2019; Guo et al., 2020). Zn was concentrated in the roots and decreased in the stems (Figure 8). This finding indicated that the reduction of Zn may inhibit stomatal opening, thereby affecting the photosynthesis of tomato leaves (Figures 2, 3). Foliar-applied Tre promoted the upward transport of Zn and increased its content in the stem. In addition, Mn and Zn have similar functions in antioxidant stress defense and protection of plant cells from reactive oxygen species (Misra et al., 2019; Yang et al., 2021b); Zhang et al. (2017) and Hashem et al. (2018) stated that Ca not only serves as a cell messenger of plant growth signals but also provides intermolecular linkages, thus maintaining the stability of cell walls and membranes. The salinity caused the Ca content to decrease, and this result was similar to research reports by Zhang et al. (2014) and Guo et al. (2020) that salt stress significantly reduces Ca concentration in plants. But it was significantly increased by the addition of Tre (Figure 8), thereby maintaining the stability of the cell membrane structure, which was consistent with the chloroplast ultrastructure results (Figure 4). As well, Chang et al. (2014) and Shahbaz et al. (2017) reported that supplying Tre reduced the adverse effects of salinity by increasing Ca uptake in rice plants. Cu is a component of certain proteins in plants and an effective electron donor and acceptor. In particular, this element also has an influence on the activation of enzymes with oxidative functions. This study revealed that salt stress decreased the Cu content in roots but increased its levels in stems and leaves. Remarkably, the application of Tre prompted the further increase in the Cu content in the stems (Figure 8). PCA further confirmed that exogenous Tre could change the distribution of mineral elements and play a positive role in maintaining ion homeostasis (Figure 9).

Na and chlorine (Cl) in high concentrations are usually the most harmful and dominant elements (Zamani et al., 2019). Plants can resist salt stress in three ways: rejection, secretion, and diluter. Tang et al. (2019) and Li et al. (2020) indicated that salinity increased the concentration of Na and decreased the concentration of K in plants. In our study, salt stress increased SK-Na in tomato plants, and this parameter was further increased by the exogenous supply of Tre (Figure 10A). These results were consistent with Shahbaz et al. (2017) and Samadi et al. (2019). One possibility is that Tre can protect the pump with specific needs by maintaining the integrity of the protein and lipid bilayers to exclude excess NaCl from the cells. The accumulation and transport of Na and K are closely related to ion channel protein activity and related gene expressions. Plants have a complex system to maintain ion homeostasis in response to ion disturbance. For example, Javaid et al. (2019); Guo et al. (2020), and Li et al. (2020) found that SOS was a Na^+^/H^+^ anti-transporter, whereas, HKT is a high-affinity K^+^ transporter. In addition, Wu (2018) displayed that NHX1 is a Na^+^, and K^+^/H^+^ exchanger in the plasma membrane and intima, and it plays an important role in regulating pH and K^+^ homeostasis and regulating the whole process from vesicle transport and cell expansion to plant development. However, ion transporters depend on the transmembrane H^+^ concentration gradient (Wu et al., 2021). Plasma membrane H^+^-ATPase is the main driving force for the secondary active transport of mineral elements in plant cells, and the H^+^ electrochemical potential gradient produced by its hydrolysis can transport Na^+^ out of cells (Allakhverdiev et al., 1999). In addition, He et al. (2014) indicated that the overexpression of the V-type proton pump subunit can improve the salt tolerance of transgenic wheat. By investigating the transcription levels of K/Na transport-related genes under salt stress, we found that the expressions of *SlSOS1*, *SlNHX1*, and *SlHKT1.1* were significantly up-regulated under S+T treatment compared with S treatment in plants. Likewise, *SlHA-A* and *SlVHA* in roots showed the same trend (Figure 10). This result explained the different ionic changes in tomato plants under salt stress and was consistent with the results for S_*K–Na*_. The results showed that Na^+^ efflux by exogenous Tre could be mediated by HKT and SOS1 in roots. In previous studies, overexpression of SOS1 or NHX1 in transgenic plants improved salt tolerance (Banjara et al., 2012; Ma et al., 2014). However, Na^+^ outflow is an energy-consuming process. Tre generated electrochemical H^+^ gradient mainly by stimulating the activities of H^+^-ATPase and V-type H^+^-ATPase in the root plasma membrane and became the driving force of Na^+^/H^+^ antiporter operation, thus transporting Na^+^ out of cells and preventing it from moving upward sequentially. In addition, Wu (2018) suggested that Na^+^ backflow from stems to roots through the phloem sap may be the main mechanism of Na^+^ transport from tissues to leaf cells in plants. We found that the expression of *SlHKT1.1* was significantly induced in stems and leaves (Figure 10), and this event was possibly involved in the recirculation of Na^+^ from the aboveground to underground parts. Shi et al. (2019) demonstrated that when Tre was combined with other forms of stress, the transcriptome was significantly different from the absence of Tre. Tre was able to trigger elongation factor thermo unstable and chitin elicitor receptor kinase receptors in a time-specific manner and these receptors lead to changes in ion flux. There are also studies that ABA signal was involved in Tre induced resistance (Yu et al., 2019; Sarkar and Sadhukhan, 2022). *AtTPS5* negatively regulated ABA signaling in Arabidopsis thaliana, which activated MYB-like transcription factors and thereby triggered the expression of HKT genes to regulate ion transport (Wang et al., 2015; Qin and Huang, 2020; Nawaz et al., 2022). These may be one of the reasons why Tre responds tightly to salinity by controlling mineral ion homeostasis. However, the molecular mechanism by which Tre regulates the influx and outflow cells of different mineral elements remains unclear.

In this study, the mechanism of salt tolerance in tomato was explored by using a hydroponic experiment and various analytical methods to clarify the effect of Tre on photosynthetic electron energy transport and mineral element distribution in tomato plants under salt stress. The regulation of mineral elements by Tre under salt stress is a key factor affecting photosynthesis. However, the enhancement may be indirect. Nevertheless, this study fills the gap in the understanding of Tre on the photosynthetic electron transport chain and ion homeostasis under salt stress, and provides specific insights and new ideas for the effectiveness of Tre in mediating salt tolerance. At the same time, it is of great significance for basic and applied plant biology and agricultural production applications.

## Conclusion

The protective functions of Tre on growth, photosynthetic performance and ion homeostasis in tomato seedlings exposed to salt stress were investigated. Our results showed that Tre could promote the growth of tomato seedlings, alleviate the degree of photoinhibition and improve photosynthetic performance under salt stress. On the one hand, Tre acts by altering stomatal opening, reducing the damage of chloroplast structure, maintaining the normal process of the Calvin cycle, and balancing the distribution of electrons. On the other hand, Tre contributes by regulating the absorption and distribution of mineral ions in different organs of tomato plants and maintaining the homeostasis of nutrient ions. Additionally, Tre can regulate key salt tolerance genes in different organs, improve the value of S_*K–Na*_, reduce excess Na^+^ toxicity in tomato plants, and improve salt tolerance. In conclusion, these results provide information on the theoretical basis for the study of the physiological functions of Tre in the context of salt stress. However, molecular biology and genomics were still needed to explore the specific role of genes in Tre biosynthesis pathway and a complex mechanism for the interaction of Tre with other signaling pathways in soil salinization.

## Data availability statement

The original contributions presented in this study are included in the article/Supplementary material, further inquiries can be directed to the corresponding author/s.

## Author contributions

YY and JX conceived and designed the research. YY, YDY, and JZ conducted the experiments. XZ, CW, and TN analyzed the data and prepared the figures and illustrations. YY wrote the manuscript. EB and JL read the manuscript and made valuable inputs. All authors read and approved the submission of the manuscript.

## Supplementary material

Supplementary Figure 1 Comparison of the Micro-Tom variety and the common variety. **(A)** Tomato plants. **(B)** Tomato fruits.

Supplementary Figure 2 Agarose gel electrophoresis of total RNA isolated from tomato roots, stems, and leaves. M: DNA Marker 2000; 1-12 bands were CK-root, CK-stem, CK-leaf, T-root, T-stem, T-leaf, S-root, S-stem, S-leaf, S+T-root, S+T-stem, and S+T-leaf, respectively. CK, control; T, 10 mM Tre; S, 150 mM NaCl; S+T, 150 mM NaCl + 10 mM Tre.

## Abbreviations

Na^+^: sodium ions
Cl^–^: chloride ions
Tre: trehalose
T6P: trehalose 6-phosphate
TPS: trehalose-6-phosphate synthase
G6P: glucose 6-phosphate
UDPG: uridine diphosphate glucose
TPP: trehalose-6-phosphate phosphatase
SEM: scanning electron microscope
TEM: transmission electron microscope
Ci: intercellular carbon dioxide concentration
Tr: transpiration rate
gs: stomata conductance
Pn: net photosynthetic rate
Rubisco: ribulose diphosphate carboxylase/oxygenase
FBPase, fructose-1: 6-bisphosphatase
TK: transketolase
GAPDH: glyceraldehyde-3-phosphate dehydrogenase
FBA: fructose-1,6-bisphosphate aldolase
V_*j*_: relative variable fluorescence intensity at the J-step
V_*i*_: relative variable fluorescence intensity at the I-step
M_*o*_: approximated initial slope of the fluorescence transient
S_*m*_: normalized total complementary area above the O-J -I-P
S_*m/t(Fm)*_: the average redox state of Q_*A*_
φP_*o*_: maximum quantum yield for primary photochemistry (at *t* = 0)
φE_*o*_: quantum yield for electron transport (at *t* = 0)
φ R_*o*_: the quantum yield of PSI final electron acceptor reduction per photon absorption
ψ_*o*_: probability that a trapped exciton moves an electron into the electron transport chain beyond Q_*A*_^–^ (at *t* = 0)
δ R_*o*_: the efficiency of electron transfer from Q_*B*_ to PSI receptor
PI_*abs*_: performance index on absorption basis
ABS/CS_*m*_: absorption flux per cross section
TRo/CS_*m*_: trapped energy flux per PSII cross section
ETo/CS_*m*_: electron transport in PSII cross section
DIo/CS_*m*_: dissipated energy flux per PSII cross section
ABS/RC: absorption flux per RC
TR_*o*_/RC: trapped energy flux per RC
ET_*o*_/RC: electron transport flux per RC
DI_*o*_/RC: dissipated energy flux per RC
RCs: active reaction centers
Na: sodium
K: potassium
Ca: calcium
Mg: magnesium
Fe: iron
Mn: manganese
Zn: zinc
Cu: copper
S_*K–Na*_: K-Na transport selectivity ratio
NaCl: sodium chloride
N: nitrogen
P: phosphorus
Cl: chlorine
H^+^: hydrogen ions
K^+^: potassium ions
RuBP: ribulose-1,5-bisphosphate
SOD: superoxide dismutase
POD: peroxidase
PBS: phosphate buffer saline
OsO_4_: osmic acid
CO_2_: carbon dioxide
OJIP curve: the chlorophyll fluorescence kinetics
PC1: the first principal component
PC2: the second principal component
SnRK1: sucrose non-fermenting-1-related protein kinase1
ROS: reactive oxygen species
PCA: principal component analysis.
