# Supplementary material for: Trehalose alleviates salt tolerance by improving photosynthetic performance and maintaining mineral ion homeostasis in tomato plants
Source: Front Plant Sci. 2022 Aug 12;13:974507. doi: 10.3389/fpls.2022.974507 (PMC9412767; doi:10.3389/fpls.2022.974507)
Supplement: Supplementary file 1 [file Table_1.DOCX]

**Supplementary material**

**Table S1** Composition of the Hoagland nutrient solution for hydroponic.

| Compounds | Concentration |
| --- | --- |
| KNO_3_ | 10mM |
| Ca(NO_3_)_2_·4H_2_O | 3 mM |
| MgSO_4_·7H_2_O | 2 mM |
| NH_4_H_2_PO_4_ | 2 mM |
| H_3_BO_3_ | 0.045 mM |
| MnCl_4_·4H_2_O | 0.01 mM |
| ZnSO_4_·7H_2_O | 0.8 μm |
| H_2_MoO_4_ | 0.4 μm |
| CuSO_4_·5H_2_O | 0.3 μm |
| FeSO_4_·7H_2_O | 0.02 mM |
| EDTA-Na_2_ | 0.02 mM |
