# Supplementary material for: Trehalose alleviates salt tolerance by improving photosynthetic performance and maintaining mineral ion homeostasis in tomato plants
Source: Front Plant Sci. 2022 Aug 12;13:974507. doi: 10.3389/fpls.2022.974507 (PMC9412767; doi:10.3389/fpls.2022.974507)
Supplement: Supplementary file 2 [file Table_2.DOCX]

**Supplementary material**

**Table S2** Formulae and terms used in the analysis of the OJIP fluorescence induction dynamics curve.

| **Formulae and terms** | **Illustrations** |
| --- | --- |
| F_o_ | Minimal recorded fluorescence intensity |
| F_m_ | Maximal recorded fluorescence intensity |
| V_j_ = (F_J_–F_o_)/(F_m_–F_o_) | Relative variable fluorescence intensity at the J-step |
| V_i_ = (F_30 ms_–F_o_)/(F_m_–F_o_) | Relative variable fluorescence intensity at the I-step |
| M_o_ = 4·(F_300 µs_–F_o_)/(F_m_–F_o_) | Approximated initial slope of the fluorescence transient |
| S_m_ = (Area)/(F_m_–F_o_) | Normalised total complementary area above the O-J-I-P transie (reflecting single-turnover Q_A_ reduction events) |
| S_m/t(Fm)_ | The average redox state of Q_A_ |
| **Yields or flux ratios** |  |
| φP_o_ = TR_o_/ABS = 1-(F_o_/F_m_) | Maximum quantum yield for primary photochemistry (at t=0) |
| φE_o_ = ET_o_/ABS = [1-(F_m_/F_m_)]·ψ_o_ | Quantum yield for electron transport (at t=0) |
| φR_o_ = 1-F_I_/F_m_ | The quantum yield of PSI final electron acceptor reduction per photon absorption |
| ψ_o_ = ET_o_/TR_o_ = 1-V_j_ | Probability that a trapped exciton moves an electron into the electron transport chain beyond Q_A_^-^ (at t=0) |
| δR_o_ = (1-V_i_)/(1-V_j_) = (F_m_-F_I_)/(F_m_-F_J_) | The efficiency of electron transfer from Q_B_ to PSI receptor |
| **Performance indexes** |  |
| PI_abs_ = (RC/ABS)·[φP_o_/(1-φP_o_)]·[ψ_o_/(1-ψ_o_)] | Performance index on absorption basis |
| **The energy flux through the unit excitation cross section** |  |
| ABS/CS_m_ ≈ F_m_ | Absorption flux per cross section |
| TRo/CS_m_ ≈ φP_o_·(ABS/CS_m_) = F_m_·[1-(F_o_/F_m_)] | Trapped energy flux per PSII cross section |
| ETo/CS_m_ ≈ F_m_·[1-( F_o_/F_m_)]·(1- V_j_) | Electron transport in PSII cross section |
| DIo/CS_m_ ≈ (ABS/CS_m_)-(TR_o_/CS_m_) | Dissipated energy flux per PSII cross section |
| **Specific energy fluxes [per Q_A_-reducing PSII reaction center (RC)]** |  |
| ABS/RC = M_o_·(1/V_j_)·(1/φP_o_) | Absorption flux per RC |
| TR_o_/RC = M_o_·(1/V_j_) | Trapped energy flux per RC |
| ET_o_/RC = M_o_·(1/V_j_)·ψ_o_ | Electron transport flux per RC |
| DI_o_/RC = (ABS/RC)-(TR_o_/RC) | Dissipated energy flux per RC |
