# Supplementary material for: Trehalose alleviates salt tolerance by improving photosynthetic performance and maintaining mineral ion homeostasis in tomato plants
Source: Front Plant Sci. 2022 Aug 12;13:974507. doi: 10.3389/fpls.2022.974507 (PMC9412767; doi:10.3389/fpls.2022.974507)
Supplement: Supplementary file 3 [file Table_3.DOCX]

**Supplementary material**

**Table S3** Primer sequences used for qRT-PCR.

| **Gene name** | **Sequence (5’-3’)** | **GenBank accession number** |
| --- | --- | --- |
| Actin | F: AATGAACTTCGTGTGGCTCCAGAG | NC_015447.3 |
|  | R: ATGGCAGGGGTGTTGAAGGTTTC |  |
| *SlHKT1.1* | F: AGGTGTTGAAGAGAACACGGA | NM_001308344 |
|  | R: GCTTCCAAGCTTTCCCTCCT |  |
| *SlNHX1* | F: CTTGGTCTGGTTCTGGTTGGAAGG | XM_010323391 |
|  | R: CCCAAACACCACTGTACTGAAGAGG |  |
| *SlSOS1* | F: TCCGCCTGCTAAATGGTGTTCAAG | NM_001247769 |
|  | R: TCCTCTCCTTCTTCCTCGCTTTCC |  |
| *SlVHA* | F: CTGATGAAGAGTGCCATTGGTGAGG | NP_001304702 |
|  | R: GGGTCTTTGCTGGGATACGATGC |  |
| *SlHA-A* | F: CCGCAAATACCGTCCTGGCATAG | NP_001234775 |
|  | R: AGCAGTATCAATCGCATCCTGGTTC |  |
| *SlRCA* | F: ACTTGCCTTGGACACTTCAGATGAC | LOC107430617 |
|  | R: TGACCTTTCCCTCCCCATATTCCC |  |
| *SlFBPase* | F: GCCACAGCAACAGCAACAACTTC | XM_015232533 |
|  | R: TGACAGCACCTTGAACTCCAGTAAG |  |
| *SlFBA* | F: GGTCTTGATGGTCTCGCAGAACG | NM_001321372 |
|  | R: GGTGGTCATTGAGAGCCTTGTAGC |  |
| *SlGAPDH* | F: ATGGAAGCATCACGAGTTGAAGGTC | XM_016099011 |
|  | R: GTGGAGCAAGGCAGTTGGTAGTG |  |
| *SlTK* | F: TTGGTGGTAGTGCTGATCTTGCTTC | XM_015200072.1 |
|  | R: CCTGCTGTCTCGTTGCCATCTG |  |
