# Supplementary material for: Trehalose alleviates salt tolerance by improving photosynthetic performance and maintaining mineral ion homeostasis in tomato plants
Source: Front Plant Sci. 2022 Aug 12;13:974507. doi: 10.3389/fpls.2022.974507 (PMC9412767; doi:10.3389/fpls.2022.974507)
Supplement: Supplementary file 4 [file Table_4.DOCX]

**Supplementary material**

**Table S4** The concentration and purity of total RNA isolated from tomato roots, stems and leaves.

| **Treatment** | **Band** | **Concentration (ng/μL)** | **OD_260_/OD_280_** |
| --- | --- | --- | --- |
| CK-root | 1 | 315.76 | 1.965 |
|  |  | 356.2 | 2.098 |
|  |  | 402.34 | 2.01 |
| CK-stem | 2 | 597.8 | 1.916 |
|  |  | 467.73 | 1.959 |
|  |  | 491.26 | 2.04 |
| CK-leaf | 3 | 344.76 | 1.989 |
|  |  | 324.55 | 2.013 |
|  |  | 348.9 | 2.025 |
| T-root | 4 | 454.44 | 2.001 |
|  |  | 493.2 | 1.976 |
|  |  | 440 | 1.955 |
| T-stem | 5 | 434.72 | 2.077 |
|  |  | 420 | 2.013 |
|  |  | 414.33 | 2.055 |
| T-leaf | 6 | 483.68 | 2.076 |
|  |  | 495.3 | 1.973 |
|  |  | 512.02 | 1.959 |
| S-root | 7 | 567.12 | 2.024 |
|  |  | 577.91 | 1.986 |
|  |  | 582.33 | 1.913 |
| S-stem | 8 | 380.76 | 1.999 |
|  |  | 414.69 | 2.04 |
|  |  | 425 | 1.971 |
| S-leaf | 9 | 513 | 2.032 |
|  |  | 537.2 | 1.96 |
|  |  | 532.47 | 1.904 |
| S+T-root | 10 | 373 | 1.956 |
|  |  | 412.31 | 2.012 |
|  |  | 398 | 1.977 |
| S+T-stem | 11 | 553.36 | 2.205 |
|  |  | 506.5 | 1.909 |
|  |  | 531.4 | 2.034 |
| S+T-leaf | 12 | 271.48 | 2.255 |
|  |  | 334.29 | 2.172 |
|  |  | 326.71 | 2.031 |

The band position is shown in Supplementary Figure S2.
