# Supplementary figures and images for: Trehalose alleviates salt tolerance by improving photosynthetic performance and maintaining mineral ion homeostasis in tomato plants
Source: Front Plant Sci. 2022 Aug 12;13:974507. doi: 10.3389/fpls.2022.974507 (PMC9412767; doi:10.3389/fpls.2022.974507)

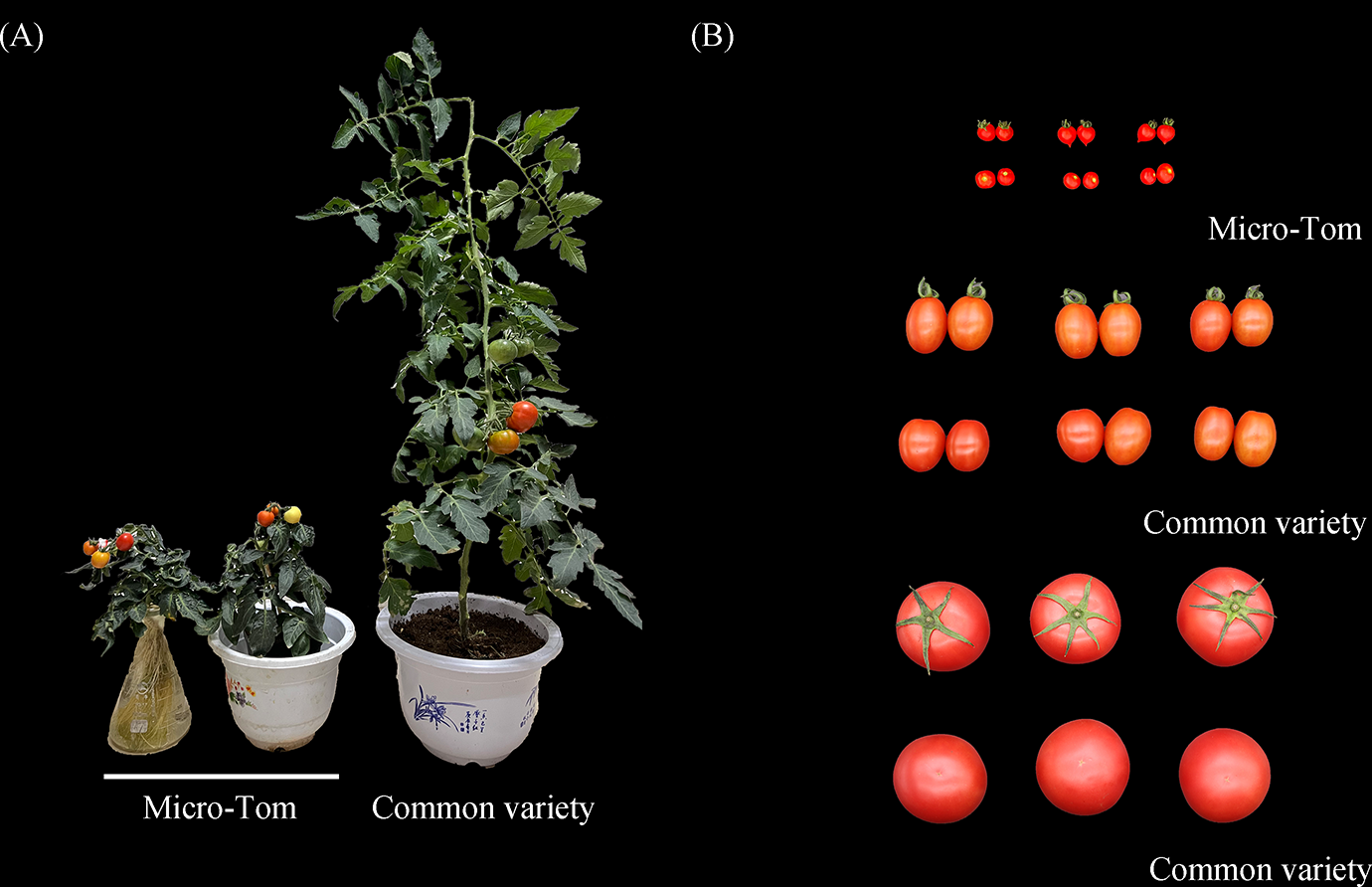

Supplement: Supplementary Figure 1 — Comparison of the Micro-Tom variety and the common variety. (A) Tomato plants. (B) Tomato fruits. [file Image_1.TIF]

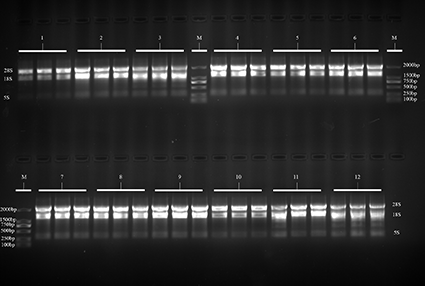

Supplement: Supplementary Figure 2 — Agarose gel electrophoresis of total RNA isolated from tomato roots, stems, and leaves. M: DNA Marker 2000; 1-12 bands were CK-root, CK-stem, CK-leaf, T-root, T-stem, T-leaf, S-root, S-stem, S-leaf, S+T-root, S+T-stem, and S+T-leaf, respectively. CK, control; T, 10 mM Tre; S, 150 mM NaCl; S+T, 150 mM NaCl + 10 mM Tre. [file Image_2.TIF]
